# Case report: Non-invasive cyto-salivary sampling and biomarker detection via ELISA versus histopathology for diagnosing oral potentially malignant disorders - Insights from a case-control study

**DOI:** 10.3389/fimmu.2024.1477477

**Published:** 2024-11-29

**Authors:** Federico Rebaudi, Alberto Rebaudi, Alfredo De Rosa, Alberto Luigi Rebaudi, Silvia Pesce, Marco Greppi, Marco Roghi, Maurizio Boggio, Simona Candiani, Emanuela Marcenaro

**Affiliations:** ^1^ Department of Experimental Medicine (DIMES), University of Genoa, Genoa, Italy; ^2^ Private Practice, Genova, Italy; ^3^ Multidisciplinary Department of Medical-Surgical and Dental Specialties, University of Campania “Luigi Vanvitelli”, Naples, Italy; ^4^ IRCCS Ospedale Policlinico San Martino, Genoa, Italy; ^5^ Department of Oral Pathology, Istituto Stomatologico Italiano, Milan, Italy; ^6^ Department of Earth, Environmental and Life Sciences (DISTAV), University of Genoa, Genoa, Italy

**Keywords:** oral potentially malignant disorders, cytobrush sampling, biomarker analysis, histopathology, immune checkpoints

## Abstract

Oral leukoplakia is classified among oral potentially malignant disorders (OPMDs) by the World Health Organization (WHO). The visual oral examination (VOE) is the most used method for identifying lesions in their early stages. Given that the diagnosis of oral cancer is often late, there is an urgent need for early detection and examination of oral lesions. Surgical biopsy represents the gold standard as a diagnostic method, but because it is invasive, it cannot be repeated for periodic checks. We report the case of a lesion on the buccal mucosa of a 65-year-old male patient with a malignant appearance. The patient underwent a novel non-invasive cyto-salivary sampling and ELISA immunoassay for tumor biomarker detection and biopsy with histopathological analysis. The rapid ELISA test results excluded signs of malignancy, providing valuable insights into the lesion’s immunophenotypic profile, which were consistent with the histopathological examination findings. This case report highlights the clinical and histopathological characteristics of a lesion with the aspect of Proliferative Verrucous Leukoplakia (PVL), emphasizing its challenging diagnosis and management. The integration of non-invasive cytobrush sampling with biomarker analysis proved valuable in detecting specific tumor biomarkers, potentially indicating ongoing tumor transformation. Monitoring these markers over time could enhance early detection and management strategies, thereby improving patient outcomes. This approach underscores the utility of non-invasive techniques in phenotyping oral lesions and supporting clinical decision-making in oral medicine.

## 1 Introduction

Leukoplakia is the most common and studied white oral lesion, classified as an oral potentially malignant disorder (OPMD). The World Health Organization (WHO) defines leukoplakia as “a predominantly white plaque of questionable risk, having excluded other known diseases or disorders that carry no increased risk for cancer.” The global prevalence of leukoplakia in the adult population is approximately 4.11% (1). Clinically, leukoplakia is diagnosed by excluding other white lesions with distinct clinicopathological characteristics. There are two main variants: homogeneous leukoplakia and non-homogeneous leukoplakia. The latter generally carries a higher risk of neoplastic transformation and exhibits varying features based on color and surface texture (2). To diagnose leukoplakia accurately, it is essential to exclude other well-defined pathologies associated with specific risk factors. These include frictional keratosis linked to persistent local trauma, tobacco pouch keratosis often found in smokers, and oral candidiasis. Experts emphasize that the initial diagnosis of leukoplakia is provisional and should be confirmed through histopathological analysis (3). A particularly aggressive form of leukoplakia is Proliferative Verrucous Leukoplakia (PVL), which is associated with a higher risk of neoplastic progression. PVL often begins as one or multiple leukoplakias that gradually enlarge, eventually merging into a single large lesion (4). Clinically, it is characterized by the gradual, continuous expansion of alterations on the oral mucosal surface, typically keratinized, which can develop varied textures and, in some cases, nodular formations that may harden over time. While there is no single histopathological definition for PVL, clinical and histological correlation is crucial for diagnosis. Accurate photographic documentation should be collected before performing a biopsy to assist the pathologist in correlating clinical and histopathological features. PVL predominantly affects females, particularly the elderly (5). It is important to note that these lesions have a tendency toward malignant progression in about 50% of cases, with carcinomas potentially developing in non-contiguous areas, particularly in the gingiva, alveolar mucosa, buccal mucosa, palate, and dorsal tongue (6). Patients with PVL should be monitored over time, and biopsies should be performed in areas that are more verrucous or nodular to exclude potential dysplasia or cancerization. Managing these rare leukoplakias is challenging, as they are often large and multifocal, complicating surgical eradication. Various treatment modalities have been described, including photodynamic therapy, laser ablation, and medical therapies, though with limited success (7, 8). Surgical removal is considered the treatment of choice, despite a recurrence rate of 71.2% (9). The purpose of this study is to correlate results obtained from a non-invasive cytobrush sampling, developed using a high-sensitivity ELISA technique for the detection of six tumor biomarkers, with findings from traditional histopathological analyses (10).

## 2 Case description

**Figure 1:**
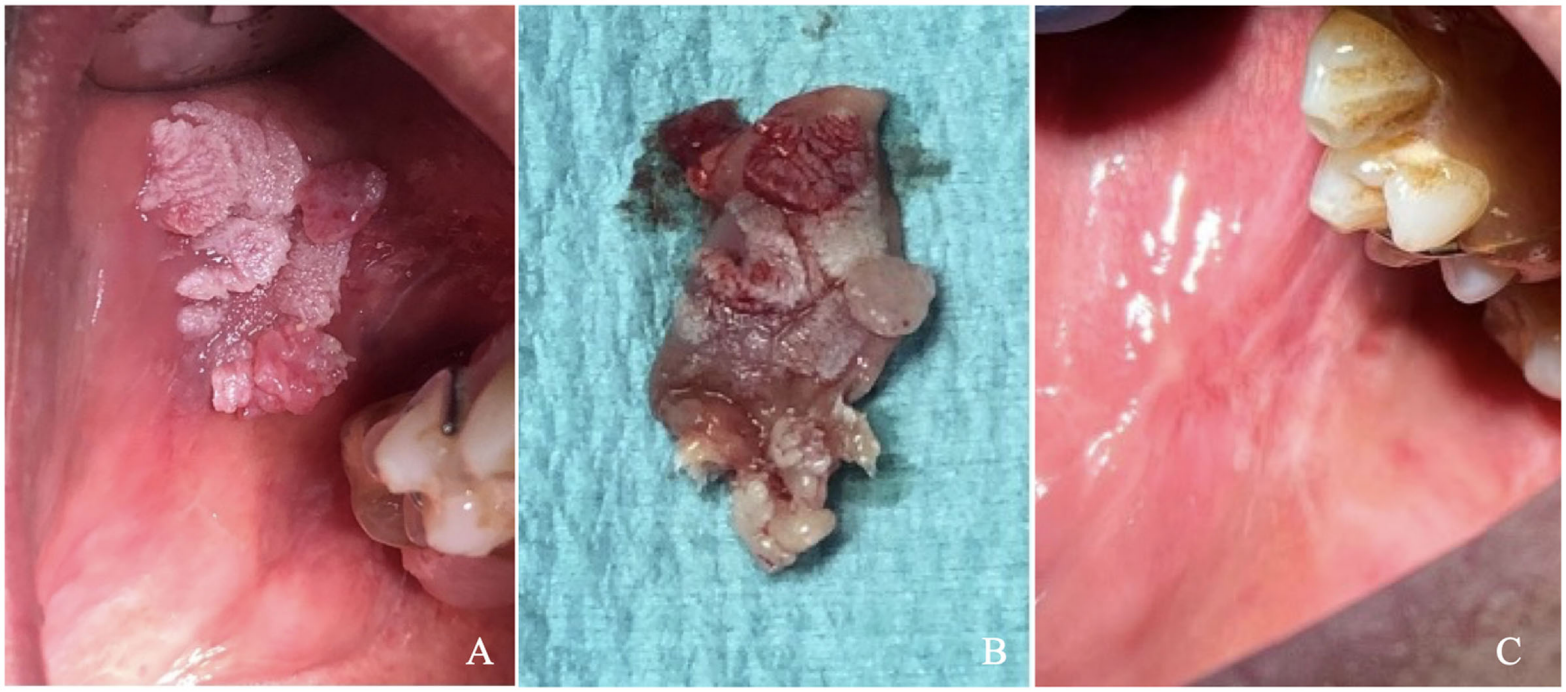
**(A)** Clinical picture showing the lesion **(B)** Excisional biopsy of the lesion immediately following surgery, **(C)** Healing of the biopsy site at 6-month follow-up.

A 65-year-old male patient presented with a cauliflower-like growth on the buccal mucosa that had developed approximately two months before the visit. Initially, the patient underestimated the lesion’s significance and delayed seeking medical attention. Concerned about the rapid growth of the lesion, he consulted his general practitioner, who ordered hematological tests and referred him for a dental examination. The patient was generally healthy, although he had a significant smoking history of 20 cigarettes per day for 30 years and was a moderate drinker, consuming about one glass of wine per meal. General clinical and hematological tests revealed no abnormalities. During the specialist examination, an extensive lesion in the fornix mucosa was noted in the upper right quadrant, corresponding to teeth positions 14, 15, 16, and 17. A removable partial resin prosthesis with metal retention clips was present in the lesion area. The patient was advised to remove the prosthesis to prevent further irritation of the lesion. Palpation of the perioral and neck glands was unremarkable, and the patient reported no additional local symptoms. Despite the presence of the partial removable prosthesis, he experienced minimal discomfort, only noting the growth of the mass. Clinical examination revealed a large lesion measuring approximately 1.5 x 1.3 cm, exhibiting a papillomatous, verruciform, and irregular appearance, as shown in **Figure 1A**. The lesion had well-defined borders, multiple new growths, and was predominantly white with some red areas. It was located on the buccal mucosa adjacent to the right maxillary premolar and first molar. The patient had no other oral or extraoral lesions. Two biopsies were performed: a non-invasive cytobrush sampling for biomarker analysis followed by an excisional biopsy of the entire lesion. After rinsing the patient’s mouth with saline, a cytosalivary sample was obtained using a cytobrush. Following the method described by Rebaudi et al., the cytobrush was gently rubbed with mild pressure and rotation over the lesion to collect cells and tissue fragments through exfoliation, taking care to avoid bleeding. The cytobrush tip was then placed into sealed Eppendorf vials, cataloged, and stored at 0-4°C before being sent to the laboratory in a refrigerated thermal box (10). Given that the lesion was less than 2 cm in size and lacked ulcerative necrotic features, it was decided to proceed with an excisional biopsy of the entire mass. After administering local anesthesia (Septodont, France), surgical excision was performed using a cold scalpel blade no. 15 (Hu Friedy, Chicago, USA). Silk sutures (Ethicon Inc., Somerville, New Jersey) were used for proper wound closure. The biopsy specimen, measuring 2 x 1.5 x 0.6 cm (**Figure 1B**), was placed in 10% formalin solution and sent to the pathologist for histopathological analysis.

### 2.1 Analysis

The analysis of biomarkers expressed by tissue fragments collected from an oral lesion through a cytobrush biopsy was conducted using two different disposable Stark Oral Screening^®^ test kits (Stark S.a.r.l.):

-Stark Oral Screening Quantitative Metabolic (REF: SOSFMTCKIT) for the detection of EGFR, p53, and Ki67.
-Stark Oral Screening Quantitative NK Time (REF: SOSBHPDQNT) for the detection of B7-H6, PD-L1, and HLA-E.

After sampling the oral mucosa, the cytobrush is immersed in a lysis buffer, and the resulting protein suspension is analyzed. This test serves as a diagnostic aid and is processed automatically by a tabletop device (Femtohunter®) using the ELISA technique. The Stark Oral Screening^®^ test is an *in vitro* diagnostic (IVD) tool that provides both qualitative and quantitative results based on a bioluminescent signal response, with a limit of detection (LOD) of 20 femtograms/microliter. Each chemiluminescent signal (S) detected by the Femtohunter® device is calibrated against the background noise (N), which is generated by non-specific luminescence on a control polyvinylidene difluoride (PVDF) membrane. The N value is subtracted from the S value detected on the PVDF membrane designated for the marker. A positive result (S - N > 0) indicates a specific signal for the target marker, confirming its presence in the sample. The positive value S is then divided by N to calculate a multiplication factor, allowing the operator to determine how many times the specific signal S is stronger than the background noise N (Signal-to-Noise ratio). The resulting S/N value is referred to as the FM (multiplication factor for the Femtohunter®) and is included in the Femtohunter® FM patient report. We observed the reproducibility of the results in previous applications of this test. This was done on a large cohort of patients with samples recovered at different time points (10).

**Figure 2:**
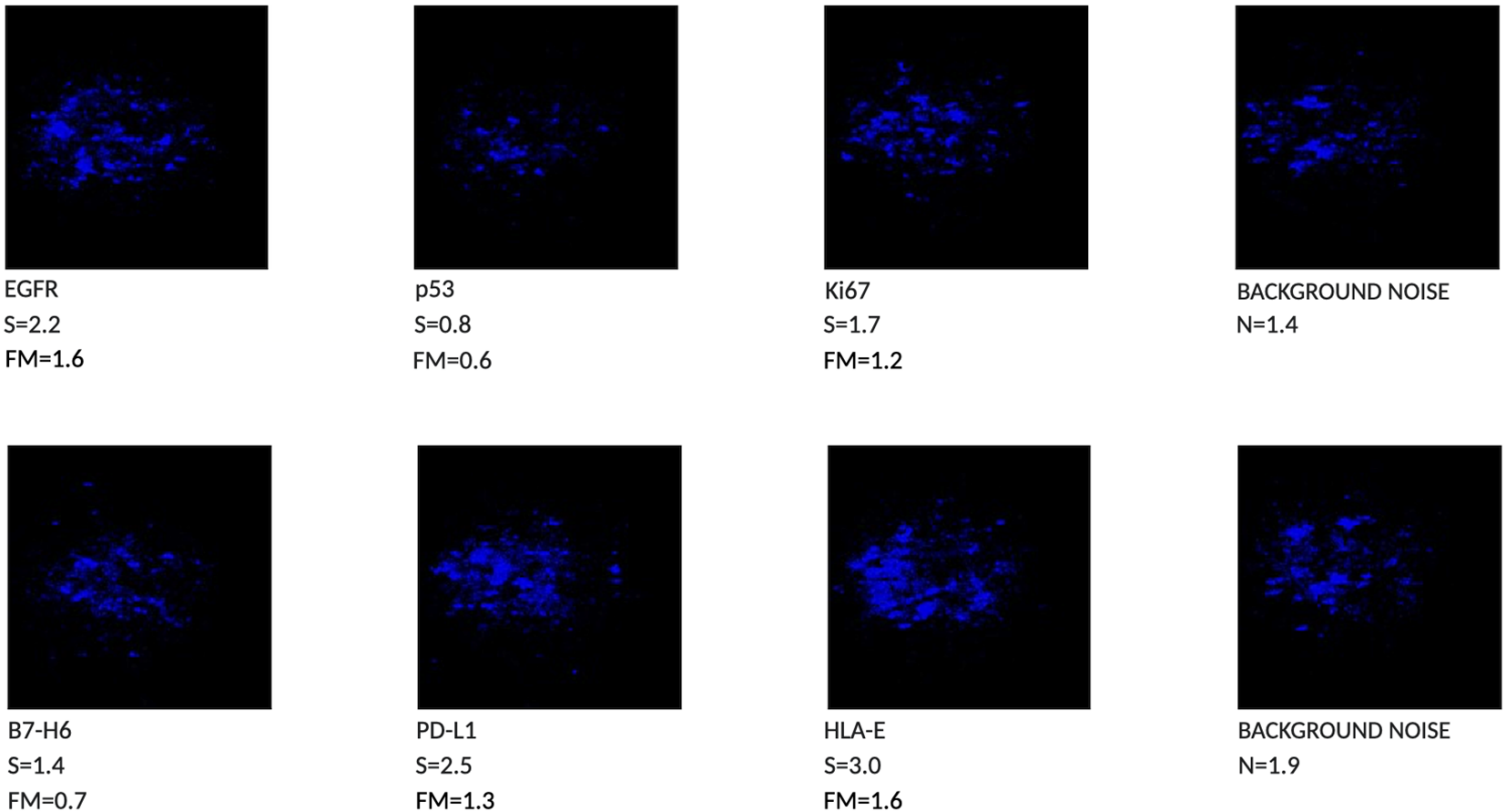
Chemiluminescent Phenotype of a lesion with aspect of Proliferative Verrucous Leukoplakia, 4 out of 6 biomarkers are positive (FM > than 1.2).

The cytobrush analysis revealed the presence of 4 out of 6 biomarkers, with Femtohunter® FM values greater than or equal to the cutoff of 1.2. Specifically, two biomarkers, p53 and B7-H6, were negative, showing FM values below 1.2. Among the positive biomarkers, EGFR and HLA-E demonstrated clear positivity, with FM values of 1.6. Ki67 and PD-L1 were weakly positive, with values of 1.2 and 1.3, respectively. Therefore, the test is considered negative for malignant tumors, as the FM values for some biomarkers were below 1.2 (**Figure 2**).

Histopathological analysis of the mucosectomy specimen revealed submucosal tissue containing adipose and stretched muscular components. The surface exhibited a keratotic exophytic lesion composed of papillomatous epithelial projections, some of which were blunt and featured keratin-rich invaginations (“tapping”), without evident fibrovascular papillae. The squamous epithelium appeared thickened, intermittently para-keratotic, and acanthotic. Epithelial ridges displayed mild atypia and rare mitotic figures, with a tendency toward convergence and fusion. Although basal hyperplasia was not observed, focal cytopathic changes suggestive of viral infection (“koilocytosis”) were present.

**Figure 3:**
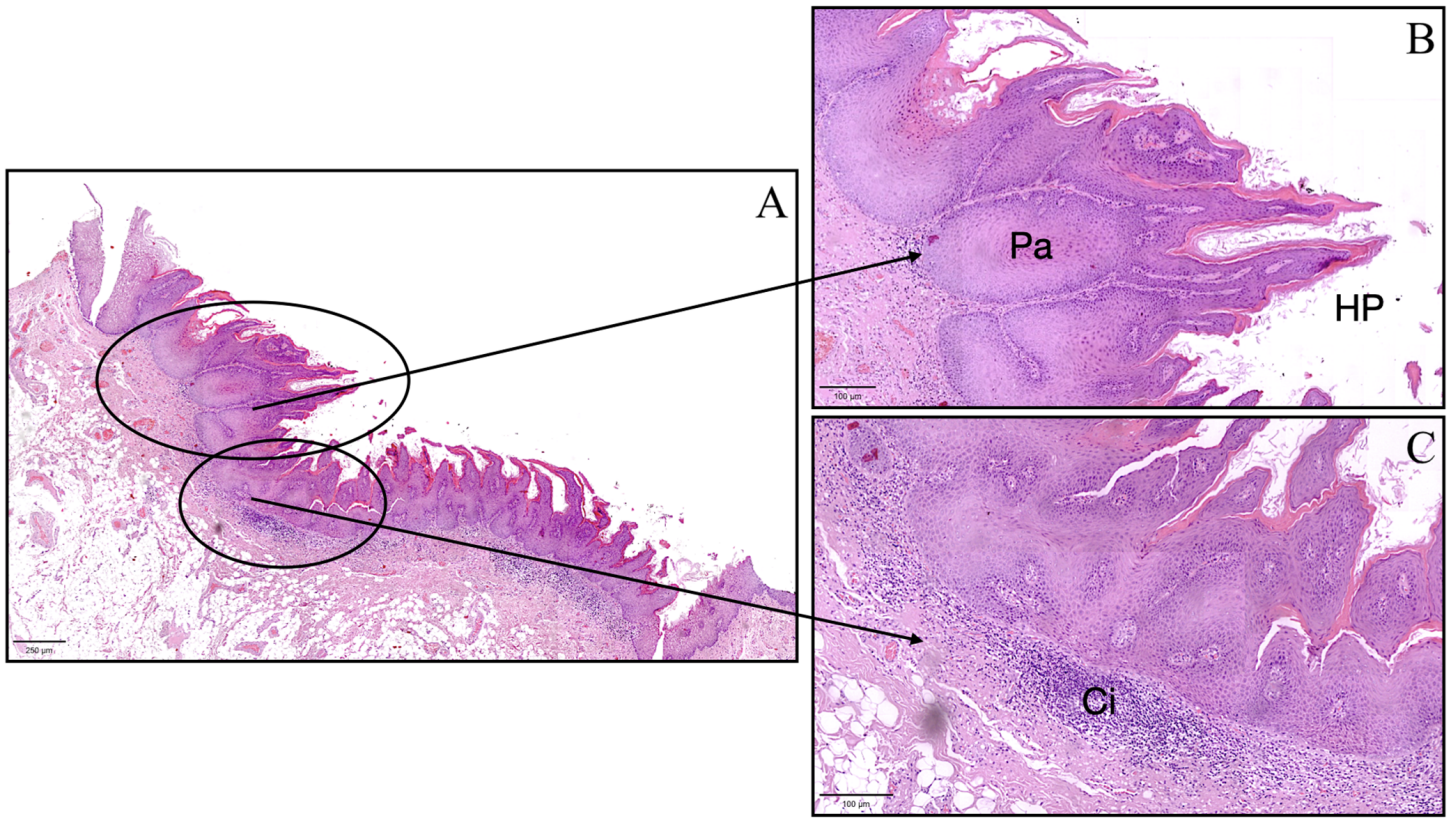
Histopathological image of the lesion with aspect of Proliferative Verrucous Leukoplakia showing pseudoinvasive aspects (Pa), hyperkeratosis and papillomatosis (HP), and chronic inflammatory infiltrate (Ci). **(A)** scale bar in **(A)** = 250 μm, scale bar in **(B**, **C)** = 100 μm.

Based on the clinical and histological findings, a diagnosis of lesion with the aspect of Proliferative Verrucous Leukoplakia (PVL) was established (**Figure 3**). A clinical follow-up at six months showed complete healing of the sampling site with no signs of local recurrence (**Figure 1C**). Despite the absence of clinical indications of recurrence, ongoing monitoring of the patient is essential to ensure that future recurrences do not occur.

## 3 Discussion

Currently, there are no scientifically endorsed systems capable of identifying lesions in their early stages of tumor development (11) other than the traditional clinical Visual Oral Examination (VOE). Surgical biopsy remains the most effective method for collecting tissue for diagnosis (12) and is considered the gold standard. However, this method is invasive and cannot be performed repeatedly for follow-up checks, particularly in cases of large lesions that cannot be completely excised. Additionally, a biopsy only reflects a portion of the lesion, depending on the surgeon’s discretion. The advantage of cytobrush sampling is its ability to detect tumor markers throughout the lesion, as sampling can encompass the entire lesion and its margins. Furthermore, since the cytobrush is a non-invasive method, it can be repeated periodically for monitoring. The case presented in this article examines the clinical and histopathological aspects of an irregular, cauliflower-like lesion with a papillomatous verruciform appearance. Proliferative Verrucous Leukoplakia (PVL) is a relatively uncommon condition with a higher prevalence in elderly women. A hallmark of this condition is its constant growth, which can occur even in non-contiguous areas, with a high estimated risk of cancerization at 50% (13). The histopathological diagnosis is challenging because there is no unique definition for this form of leukoplakia; clinical and histological correlation is essential for an accurate diagnosis (5, 6). The role of human papillomavirus (HPV) in this type of lesion is controversial. Some studies, such as those by Palefsky et al. (1995) (14), have detected HPV in many PVL cases, while others have found no evidence of HPV in PVL (15, 16). In the case described, histopathological analysis revealed the presence of HPV, characterized by focal, attenuated cytopathic features suggestive of viral infection, known as “koilocytosis.” Since this lesion could not be classified as PVL due to incomplete clinical and histopathological correspondence, it was categorized as a lesion with aspects of PVL. The Femtohunter^®^ is an automatic ELISA developer device that performs chemiluminescence analysis on samples taken by cytobrush and submitted to the Stark Oral Screening^®^ IVD test. Results are provided as a graphical image (**Figure 2**), along with analytical data of the markers, and a patient report is printed. The Stark Oral Screening^®^ test is a patient-side *in vitro* diagnostic (IVD) and quantitative test based on bioluminescent signal response (10). The biomarker analysis of the cytobrush revealed the presence of 4 out of 6 positive biomarkers. These biomarkers were considered positive because their FM values were greater than or equal to the cutoff of 1.2. Two biomarkers, p53 (FM 0.6) and B7-H6 (FM 0.7), were negative. Among the positive biomarkers, EGFR and HLA-E showed clear positivity, with FM values of 1.6. Ki67 and PD-L1 exhibited weak positivity, with values of 1.2 and 1.3, respectively. The test results indicate a negative outcome for a malignant tumor, as a positive diagnosis requires all six biomarkers to have FM values greater than 1.2 (10). Analyzing the Individual Markers in Detail:

-PD-L1 is a transmembrane protein expressed in various types of tumors, including Oral Squamous Cell Carcinoma (OSCC) (17, 18). When bound to the inhibitory checkpoint PD-1 (originally identified on T cells and more recently on NK cells), PD-L1 compromises the ability of cytotoxic immune cells to eliminate the tumor (19–21). Pharmacological treatments exist that inhibit the PD-1/PD-L1 axis, leading to improved survival in OSCC patients (22). The presence of PD-L1 in OPMDs has been documented in several studies (23, 24). Notably, a study by Dave et al. (25) demonstrated that PD-L1 could be present in a precancerous lesion even years before potential malignant transformation, aligning with our findings of weak but detectable expression of this marker (FM 1.3).
-Moderately elevated expression of Ki67 has been reported by Fettig et al. (2000) (26) in a study analyzing 10 cases of PVL. In this study, Ki67 expression was not correlated with the level of epithelial alterations. In another study involving 12 patients, Gouvea et al. (2010) (27) found Ki67 expression in PVL lesions. Our case also exhibited low positivity for this marker, consistent with the aforementioned studies (FM 1.2).
-EGFR is a transmembrane receptor that regulates signaling involved in cell proliferation and differentiation. Numerous studies have shown that leukoplakia often exhibits overexpression of EGFR, which correlates with a higher risk of malignant transformation (28). This suggests that EGFR could serve as a biological marker for identifying high-risk subgroups of OPMDs (29). In our case, we observed high levels of EGFR expression (FM 1.6).
-Altered expression of HLA-E, the ligand for the inhibitory checkpoint NKG2A (found on NK cells), has been noted in oral inflammatory/pre-tumoral conditions (17, 24, 30). The expression of molecules such as HLA-E and PD-L1 is independent of the oral lesion’s histopathological grade; levels of these molecules are comparable in OPMDs and oral squamous cell carcinomas (24), with FM 1.6 in our case.
-B7-H6 (31), a member of the B7 family of immune modulators, was originally identified as a ligand for NKp30 (32), a receptor on NK cells. Expression of B7-H6 on tumor cell surfaces can enhance susceptibility to NK cell-mediated attacks. Several studies suggest that a soluble form of B7-H6 could be released by tumor cells, affecting NKp30 surface expression and preventing effective anti-tumor activity (33, 34). B7-H6 is expressed in various tumor types but absent in normal tissues, aligning with its sub-threshold expression in this lesion with precancerous characteristics (FM 0.7).
-Alteration or mutation of the p53 gene is among the most common events in human carcinogenesis (35). The mutated protein is not easily degradable, accumulating in cancer cells and leading to immunohistochemical overexpression, which is a marker of poor prognosis. Overexpression of p53 may decrease the sensitivity of tumor cells to chemotherapy, indicating an increased risk of progression to oral cancer among OPMDs (34, 36). In our case, both B7-H6 and p53 levels were low, with p53 at FM 0.6, below the cutoff value of 1.2.

In conclusion, our findings indicate that 4 out of 6 markers are positive, suggesting that OPMDs may already express tumor markers, in contrast to healthy mucosal tissue where these markers are generally absent (10). Our approach utilizes fresh tissue fragments through exfoliation and a highly sensitive ELISA, allowing us to identify biomarkers that might otherwise remain undetected. While immunohistochemistry can provide valuable information, it typically struggles to visualize biomarkers at such low expression levels. This underscores the potential of our approach for early identification of at-risk patients, allowing for proactive management strategies.

Furthermore, we noted that in inflamed tissues, initial marker expression was typically elevated but decreased as inflammation resolved. This variability highlights the necessity of considering the dynamic nature of inflammation when interpreting biomarker results across different contexts. By recognizing these patterns, we can better differentiate between precancerous states and normal tissue, enhancing our understanding of oral pathologies. By utilizing this innovative screening method, we can enhance our ability to monitor patients over time, allowing for the timely detection of changes that may signify malignant transformation.

Monitoring the expression of these biomarkers over time could provide valuable insights into ongoing tumor transformation, facilitating timely intervention. Vigilant monitoring is essential for ensuring early diagnosis, which significantly improves patient prognosis.

Regular assessment using this non-invasive technique can empower clinicians to make informed decisions regarding patient care, ultimately leading to better treatment outcomes.

Furthermore, the use of a rapid, non-invasive system that is well-tolerated by patients for detecting tumor biomarkers could serve as an effective screening tool. This approach would aid in the phenotyping of oral lesions and provide critical information for improved management and treatment outcomes. Incorporating such screening methods into routine clinical practice could revolutionize our approach to managing OPMDs, fostering a proactive rather than reactive strategy in oral cancer prevention.

## Data Availability

The raw data supporting the conclusions of this article will be made available by the authors, without undue reservation.
